# [6-(4-Bromo­phen­yl)-2,2′-bipyridine-κ^2^ *N*,*N*′]bis­(triphenyl­phosphane-κ*P*)copper(I) tetra­fluoridoborate

**DOI:** 10.1107/S1600536811029515

**Published:** 2011-08-02

**Authors:** Yan-Ru Lin, Jun-Sheng Huang, Ming-Hua Zhong

**Affiliations:** a Department of Chemistry, Hanshan Normal University, Chaozhou 521041, People’s Republic of China

## Abstract

The title compound, [Cu(C_16_H_11_BrN_2_)(C_18_H_15_P)_2_]BF_4_, is composed of one Cu^I^ atom, one 6-(4-bromo­phen­yl)-2,2′-bipyridine (*L*) ligand, two triphenyl­phosphane mol­ecules and one tetra­fluoridoborate anion. The Cu^I^ ion is four-coordinated in a distorted tetra­hedral configuration by two N atoms from *L* and two P atoms from triphenyl­phosphane ligands. In the *L* ligand, the two pyridine rings are not coplanar; the mean planes making a dihedral angle of 15.3 (5)°. In the crystal, the ions are linked by weak C—H⋯F inter­actions.

## Related literature

For background to Cu^I^ complexes, see: Wang *et al.* (2010 ▶). For related structures, see: Engelhardt *et al.* (1985 ▶); Kirchhoff *et al.* (1985 ▶); Navarro *et al.* (2008 ▶); Peng (2010 ▶).
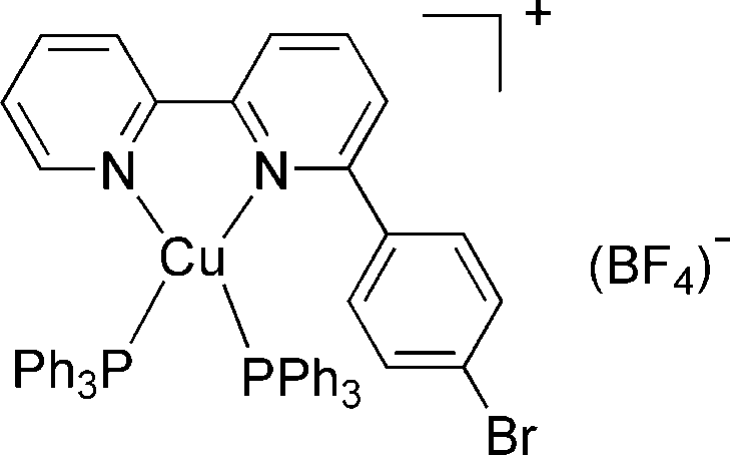


## Experimental

### 

#### Crystal data

[Cu(C_16_H_11_BrN_2_)(C_18_H_15_P)_2_]BF_4_
*M* *_r_* = 986.07
Monoclinic, 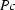

*a* = 9.992 (1) Å
*b* = 11.2591 (11) Å
*c* = 20.883 (2) Å
β = 98.658 (1)°
*V* = 2322.6 (4) Å^3^
*Z* = 2
Mo *K*α radiation
μ = 1.45 mm^−1^
*T* = 298 K
0.3 × 0.2 × 0.1 mm

#### Data collection

Bruker SMART CCD area-detector diffractometer
Absorption correction: multi-scan (*SADABS*; Bruker, 2001 ▶) *T*_min_ = 0.520, *T*_max_ = 0.758
11516 measured reflections
6547 independent reflections
4275 reflections with *I* > 2σ(*I*)
*R*_int_ = 0.066

#### Refinement

*R*[*F* ^2^ > 2σ(*F* ^2^)] = 0.056
*wR*(*F* ^2^) = 0.142
*S* = 0.95
6547 reflections
568 parameters
2 restraints
H-atom parameters constrained
Δρ_max_ = 0.47 e Å^−3^
Δρ_min_ = −0.42 e Å^−3^
Absolute structure: Flack (1983 ▶), 1482 Friedel pairs
Flack parameter: 0.010 (13)

### 

Data collection: *SMART* (Bruker, 1998 ▶); cell refinement: *SAINT* (Bruker, 1998 ▶); data reduction: *SAINT*; program(s) used to solve structure: *SHELXS97* (Sheldrick, 2008 ▶); program(s) used to refine structure: *SHELXL97* (Sheldrick, 2008 ▶); molecular graphics: *SHELXTL* (Sheldrick, 2008 ▶); software used to prepare material for publication: *SHELXTL*.

## Supplementary Material

Crystal structure: contains datablock(s) global, I. DOI: 10.1107/S1600536811029515/zq2111sup1.cif

Structure factors: contains datablock(s) I. DOI: 10.1107/S1600536811029515/zq2111Isup2.hkl

Additional supplementary materials:  crystallographic information; 3D view; checkCIF report

## Figures and Tables

**Table:** 

Cu1—N1	2.095 (7)
Cu1—N2	2.178 (6)
Cu1—P2	2.2648 (19)
Cu1—P1	2.276 (2)

**Table:** 

N1—Cu1—N2	78.7 (3)
N1—Cu1—P2	105.7 (2)
N2—Cu1—P2	126.24 (16)
N1—Cu1—P1	107.35 (18)
N2—Cu1—P1	98.60 (16)
P2—Cu1—P1	128.29 (9)

**Table 2:** Hydrogen-bond geometry (Å, °)

*D*—H⋯*A*	*D*—H	H⋯*A*	*D*⋯*A*	*D*—H⋯*A*
C26—H26⋯F2	0.93	2.53	3.185 (12)	127
C27—H27⋯F3	0.93	2.48	3.356 (11)	158

**Fig. 1.**
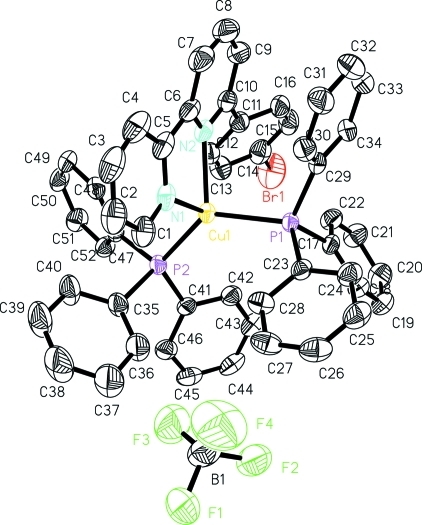
The molecular structure of the title compound. Displacement ellipsoids are drawn at the 30% probability level, and all hydrogen atoms are omitted for clarity.

## supplementary crystallographic information

### Comment

Copper(I) complexes with diimine and phosphane ligands have attracted much attention for their rich photophysical properties and diversity coordination geometry (Engelhardt *et al.*, 1985; Kirchhoff *et al.*, 1985; Navarro *et al.*, 2008; Wang *et al.*, 2010). According to the size of diimine and phosphane ligands, these complexes can adopt three- and four-coordination modes around the metal center. Peng (2010) previously reported a three-coordinated copper(I) complex with 6-(4-bromo)phenyl-2,2'-bipyridine, here we report its related four-coordinated species.

The crystal structure of the title compound is depicted in Fig. 1. The Cu^I^ ion is four-coordinated in a distorted tetrahedral geometry by two N atoms from 6-(4-bromo)phenyl-2,2'-bipyridine (*L*) and two P atoms from triphenylphosphane molecules. The coordination bond angles around the Cu atom vary from 78.7 (3)° (N1—Cu1—N2) to 128.29 (9)° (P1—Cu1—P1). The two Cu—P bond distances of 2.265 (2) and 2.276 (2) Å are very similar while the Cu—N bond distance is slightly longer with the N atom of the substituted pyridine ring (2.178 (6) Å) than with the other one (2.095 (7) Å). These bond distances are within the normal ranges of related complexes (Engelhardt *et al.*, 1985; Wang *et al.*, 2010). In addition, the two pyridine rings in ligand *L* are not coplanar, the mean planes exhibit a dihedral angle of 15.3 (5) °. In the crystal, the ions are linked by weak C-H···F interactions (Table 1).

### Experimental

The ligand 6-(4-bromophenyl)-2,2'-bipyridine (*L*) was prepared by a literature method (Wang *et al.*, 2010). A mixture of [Cu(CH_3_CN)_4_]BF_4_ (100 mg, 0.32 mmol) and *L* (99 mg, 0.32 mmol) in dichloromethane (20 ml) was stirred under nitrogen atmosphere at room temperature for 2 h. Then triphenylphosphane (170 mg, 0.64 mmol) was added kept stirring for 2 h. The solvents were removed and the solid residue was afforded. Yellow single crystals suitable for X-ray diffraction were obtained from the solution of dichloromethane by vapor diffusion with diethyl ether (yield: 82%). Analysis calculated for [Cu(C_16_H_11_N_2_Br)(C_18_H_15_P)_2_].(BF_4_): C 63.29, H 4.19, N 2.84%; Found: C 63.38, H 4.03, 2.91%.

### Refinement

All H atoms were positioned geomertrically and treated as riding with C—H = 0.93 Å and *U*_iso_(H) = 1.2 *U*_eq_(C).

### Crystal data

**Table:**

[Cu(C_16_H_11_BrN_2_)(C_18_H_15_P)_2_]BF_4_	*F*(000) = 1004
*M**_r_* = 986.07	*D*_x_ = 1.41 Mg m^−^^3^
Monoclinic, *P**c*	Mo *K*α radiation, λ = 0.71073 Å
Hall symbol: P -2yc	Cell parameters from 548 reflections
*a* = 9.992 (1) Å	θ = 2.5–26.3°
*b* = 11.2591 (11) Å	µ = 1.45 mm^−^^1^
*c* = 20.883 (2) Å	*T* = 298 K
β = 98.658 (1)°	Block, yellow
*V* = 2322.6 (4) Å^3^	0.3 × 0.2 × 0.1 mm
*Z* = 2	

### Data collection

**Table:**

Bruker SMART CCD area-detector diffractometer	6547 independent reflections
Radiation source: fine-focus sealed tube	4275 reflections with *I* > 2σ(*I*)
graphite	*R*_int_ = 0.066
φ and ω scans	θ_max_ = 25.0°, θ_min_ = 1.8°
Absorption correction: multi-scan (*SADABS*; Bruker, 2001)	*h* = −11→11
*T*_min_ = 0.520, *T*_max_ = 0.758	*k* = −13→13
11516 measured reflections	*l* = −22→24

### Refinement

**Table:**

Refinement on *F*^2^	Secondary atom site location: difference Fourier map
Least-squares matrix: full	Hydrogen site location: inferred from neighbouring sites
*R*[*F*^2^ > 2σ(*F*^2^)] = 0.056	H-atom parameters constrained
*wR*(*F*^2^) = 0.142	*w* = 1/[σ^2^(*F*_o_^2^) + (0.0756*P*)^2^] where *P* = (*F*_o_^2^ + 2*F*_c_^2^)/3
*S* = 0.95	(Δ/σ)_max_ = 0.001
6547 reflections	Δρ_max_ = 0.47 e Å^−^^3^
568 parameters	Δρ_min_ = −0.42 e Å^−^^3^
2 restraints	Absolute structure: Flack (1983), 1482 Friedel pairs
Primary atom site location: structure-invariant direct methods	Flack parameter: 0.010 (13)

### Special details

**Table:**

Geometry. All e.s.d.'s (except the e.s.d. in the dihedral angle between two l.s. planes) are estimated using the full covariance matrix. The cell e.s.d.'s are taken into account individually in the estimation of e.s.d.'s in distances, angles and torsion angles; correlations between e.s.d.'s in cell parameters are only used when they are defined by crystal symmetry. An approximate (isotropic) treatment of cell e.s.d.'s is used for estimating e.s.d.'s involving l.s. planes.
Refinement. Refinement of *F*^2^ against ALL reflections. The weighted *R*-factor *wR* and goodness of fit *S* are based on *F*^2^, conventional *R*-factors *R* are based on *F*, with *F* set to zero for negative *F*^2^. The threshold expression of *F*^2^ > σ(*F*^2^) is used only for calculating *R*-factors(gt) *etc*. and is not relevant to the choice of reflections for refinement. *R*-factors based on *F*^2^ are statistically about twice as large as those based on *F*, and *R*- factors based on ALL data will be even larger.

### Fractional atomic coordinates and isotropic or equivalent isotropic displacement parameters (Å^2^)

**Table:**

	*x*	*y*	*z*	*U*_iso_*/*U*_eq_	
Cu1	0.08269 (9)	0.25186 (7)	0.57323 (5)	0.0422 (2)	
Br1	−0.04461 (17)	−0.19694 (14)	0.33891 (7)	0.1352 (6)	
F1	0.7674 (8)	0.7221 (7)	0.7779 (3)	0.126 (2)	
F2	0.6759 (7)	0.7004 (9)	0.6758 (4)	0.148 (3)	
F3	0.5916 (9)	0.6051 (8)	0.7486 (4)	0.163 (3)	
F4	0.5706 (12)	0.7991 (11)	0.7398 (7)	0.241 (6)	
N1	0.0407 (8)	0.3522 (6)	0.6523 (3)	0.0611 (19)	
N2	−0.1375 (6)	0.2447 (5)	0.5597 (3)	0.0544 (16)	
P1	0.10772 (18)	0.38136 (17)	0.49217 (9)	0.0416 (5)	
P2	0.22605 (18)	0.10660 (16)	0.61565 (9)	0.0457 (5)	
B1	0.6459 (17)	0.7107 (15)	0.7390 (9)	0.105 (4)	
C1	0.1291 (11)	0.3954 (7)	0.7000 (4)	0.075 (3)	
H1	0.2208	0.3814	0.6998	0.090*	
C2	0.0894 (14)	0.4620 (9)	0.7510 (5)	0.089 (3)	
H2	0.1529	0.4895	0.7849	0.107*	
C3	−0.0366 (15)	0.4833 (10)	0.7491 (6)	0.095 (3)	
H3	−0.0640	0.5291	0.7818	0.114*	
C4	−0.1330 (12)	0.4414 (9)	0.7008 (6)	0.092 (3)	
H4	−0.2242	0.4582	0.7007	0.110*	
C5	−0.0910 (10)	0.3729 (7)	0.6518 (4)	0.069 (2)	
C6	−0.1862 (9)	0.3256 (7)	0.5971 (4)	0.061 (2)	
C7	−0.3173 (11)	0.3699 (8)	0.5832 (6)	0.082 (3)	
H7	−0.3474	0.4282	0.6091	0.099*	
C8	−0.3996 (11)	0.3271 (9)	0.5315 (6)	0.088 (3)	
H8	−0.4882	0.3545	0.5218	0.105*	
C9	−0.3525 (9)	0.2435 (8)	0.4935 (5)	0.072 (2)	
H9	−0.4087	0.2142	0.4574	0.086*	
C10	−0.2234 (9)	0.2027 (7)	0.5082 (4)	0.062 (2)	
C11	−0.1732 (8)	0.1095 (7)	0.4682 (4)	0.058 (2)	
C12	−0.0876 (8)	0.0190 (7)	0.4935 (4)	0.058 (2)	
H12	−0.0551	0.0191	0.5377	0.070*	
C13	−0.0491 (9)	−0.0706 (8)	0.4561 (4)	0.070 (2)	
H13	0.0085	−0.1304	0.4744	0.084*	
C14	−0.0977 (10)	−0.0706 (8)	0.3902 (5)	0.078 (3)	
C15	−0.1830 (10)	0.0149 (9)	0.3641 (5)	0.079 (3)	
H15	−0.2167	0.0127	0.3201	0.095*	
C16	−0.2204 (9)	0.1042 (8)	0.4012 (5)	0.070 (3)	
H16	−0.2785	0.1631	0.3821	0.084*	
C17	0.1686 (7)	0.3258 (6)	0.4196 (4)	0.0480 (18)	
C18	0.2859 (8)	0.3657 (7)	0.3995 (4)	0.060 (2)	
H18	0.3323	0.4296	0.4206	0.073*	
C19	0.3359 (10)	0.3115 (9)	0.3482 (5)	0.078 (3)	
H19	0.4167	0.3371	0.3358	0.094*	
C20	0.2633 (11)	0.2186 (9)	0.3156 (5)	0.082 (3)	
H20	0.2955	0.1828	0.2808	0.098*	
C21	0.1480 (10)	0.1800 (8)	0.3336 (5)	0.074 (3)	
H21	0.1002	0.1177	0.3116	0.089*	
C22	0.1013 (9)	0.2335 (7)	0.3848 (4)	0.064 (2)	
H22	0.0207	0.2064	0.3968	0.077*	
C23	0.2282 (7)	0.4979 (6)	0.5200 (4)	0.0507 (19)	
C24	0.2378 (8)	0.6019 (7)	0.4860 (5)	0.067 (2)	
H24	0.1804	0.6155	0.4473	0.081*	
C25	0.3355 (10)	0.6873 (7)	0.5106 (6)	0.078 (3)	
H25	0.3421	0.7580	0.4883	0.094*	
C26	0.4193 (9)	0.6667 (8)	0.5663 (6)	0.081 (3)	
H26	0.4861	0.7220	0.5808	0.097*	
C27	0.4083 (8)	0.5668 (8)	0.6017 (5)	0.075 (3)	
H27	0.4634	0.5557	0.6412	0.090*	
C28	0.3135 (8)	0.4819 (7)	0.5777 (4)	0.063 (2)	
H28	0.3073	0.4123	0.6011	0.075*	
C29	−0.0478 (7)	0.4661 (6)	0.4681 (4)	0.0501 (18)	
C30	−0.0807 (9)	0.5535 (8)	0.5104 (5)	0.069 (2)	
H30	−0.0203	0.5721	0.5475	0.082*	
C31	−0.2001 (10)	0.6110 (9)	0.4977 (5)	0.085 (3)	
H31	−0.2218	0.6661	0.5277	0.101*	
C32	−0.2898 (11)	0.5924 (10)	0.4433 (6)	0.092 (3)	
H32	−0.3695	0.6361	0.4350	0.110*	
C33	−0.2598 (9)	0.5072 (10)	0.4009 (5)	0.081 (3)	
H33	−0.3219	0.4908	0.3641	0.097*	
C34	−0.1374 (8)	0.4439 (7)	0.4116 (4)	0.062 (2)	
H34	−0.1166	0.3884	0.3816	0.075*	
C35	0.3299 (9)	0.1538 (7)	0.6912 (4)	0.067 (3)	
C36	0.4395 (11)	0.2260 (8)	0.6891 (5)	0.088 (3)	
H36	0.4599	0.2487	0.6488	0.106*	
C37	0.5204 (15)	0.2660 (11)	0.7434 (7)	0.129 (5)	
H37	0.6021	0.3044	0.7414	0.155*	
C38	0.4735 (17)	0.2462 (12)	0.8019 (7)	0.140 (6)	
H38	0.5212	0.2781	0.8397	0.169*	
C39	0.3608 (15)	0.1820 (11)	0.8054 (6)	0.127 (5)	
H39	0.3320	0.1702	0.8453	0.153*	
C40	0.2885 (11)	0.1339 (9)	0.7502 (5)	0.091 (3)	
H40	0.2119	0.0883	0.7527	0.109*	
C41	0.3475 (7)	0.0474 (6)	0.5679 (4)	0.0516 (19)	
C42	0.3197 (8)	0.0550 (7)	0.5007 (4)	0.060 (2)	
H42	0.2400	0.0910	0.4813	0.073*	
C43	0.4085 (10)	0.0098 (7)	0.4627 (5)	0.068 (2)	
H43	0.3860	0.0145	0.4179	0.082*	
C44	0.5269 (10)	−0.0411 (7)	0.4878 (6)	0.076 (3)	
H44	0.5856	−0.0712	0.4613	0.091*	
C45	0.5581 (9)	−0.0468 (7)	0.5546 (6)	0.073 (3)	
H45	0.6398	−0.0808	0.5729	0.088*	
C46	0.4728 (8)	−0.0043 (7)	0.5942 (5)	0.064 (2)	
H46	0.4970	−0.0092	0.6389	0.077*	
C47	0.1366 (8)	−0.0228 (6)	0.6396 (3)	0.0494 (19)	
C48	0.0138 (9)	−0.0083 (7)	0.6623 (4)	0.060 (2)	
H48	−0.0175	0.0682	0.6681	0.072*	
C49	−0.0630 (9)	−0.1035 (8)	0.6764 (4)	0.068 (2)	
H49	−0.1451	−0.0923	0.6914	0.081*	
C50	−0.0145 (10)	−0.2154 (8)	0.6676 (5)	0.074 (3)	
H50	−0.0654	−0.2807	0.6767	0.089*	
C51	0.1040 (10)	−0.2334 (7)	0.6464 (5)	0.070 (2)	
H51	0.1343	−0.3105	0.6416	0.084*	
C52	0.1816 (8)	−0.1381 (6)	0.6316 (4)	0.059 (2)	
H52	0.2633	−0.1512	0.6164	0.070*	

### Atomic displacement parameters (Å^2^)

**Table:**

	*U*^11^	*U*^22^	*U*^33^	*U*^12^	*U*^13^	*U*^23^
Cu1	0.0501 (4)	0.0413 (4)	0.0362 (4)	0.0021 (4)	0.0101 (3)	0.0013 (4)
Br1	0.2025 (16)	0.1209 (10)	0.0880 (9)	−0.0089 (10)	0.0405 (9)	−0.0422 (9)
F1	0.134 (6)	0.156 (6)	0.082 (5)	−0.016 (4)	−0.004 (4)	−0.012 (4)
F2	0.112 (5)	0.251 (9)	0.082 (5)	−0.064 (5)	0.017 (4)	−0.012 (5)
F3	0.167 (7)	0.177 (8)	0.141 (7)	−0.058 (6)	0.011 (6)	0.019 (6)
F4	0.208 (12)	0.225 (12)	0.288 (16)	0.094 (10)	0.034 (10)	0.002 (11)
N1	0.087 (6)	0.060 (4)	0.040 (4)	0.007 (4)	0.021 (4)	0.003 (3)
N2	0.064 (4)	0.047 (3)	0.056 (4)	0.001 (3)	0.023 (3)	0.011 (3)
P1	0.0442 (11)	0.0412 (9)	0.0391 (11)	−0.0030 (8)	0.0052 (8)	0.0051 (9)
P2	0.0494 (11)	0.0442 (10)	0.0426 (11)	−0.0022 (9)	0.0043 (8)	0.0082 (9)
B1	0.092 (11)	0.111 (11)	0.114 (13)	−0.031 (9)	0.022 (10)	−0.005 (9)
C1	0.105 (7)	0.069 (5)	0.054 (6)	−0.001 (5)	0.020 (5)	−0.010 (5)
C2	0.136 (10)	0.073 (6)	0.062 (7)	−0.006 (7)	0.025 (7)	−0.013 (5)
C3	0.139 (11)	0.085 (7)	0.074 (8)	−0.007 (8)	0.054 (8)	−0.009 (6)
C4	0.109 (8)	0.086 (7)	0.091 (8)	−0.003 (6)	0.051 (7)	−0.005 (7)
C5	0.089 (7)	0.057 (5)	0.069 (6)	0.005 (5)	0.039 (5)	0.009 (5)
C6	0.064 (6)	0.055 (5)	0.071 (6)	0.007 (4)	0.034 (5)	0.013 (5)
C7	0.086 (8)	0.075 (6)	0.096 (8)	0.020 (6)	0.045 (6)	0.011 (6)
C8	0.071 (6)	0.089 (7)	0.108 (9)	0.013 (6)	0.030 (6)	0.018 (7)
C9	0.061 (6)	0.073 (6)	0.084 (7)	0.003 (5)	0.017 (5)	0.014 (5)
C10	0.060 (6)	0.056 (4)	0.070 (6)	−0.005 (4)	0.014 (5)	0.022 (5)
C11	0.057 (5)	0.063 (5)	0.055 (5)	−0.011 (4)	0.007 (4)	0.004 (4)
C12	0.065 (5)	0.059 (5)	0.050 (5)	−0.007 (4)	0.005 (4)	0.002 (4)
C13	0.084 (6)	0.068 (5)	0.058 (6)	0.002 (5)	0.009 (5)	−0.011 (5)
C14	0.091 (7)	0.072 (6)	0.072 (7)	−0.022 (5)	0.015 (5)	−0.004 (5)
C15	0.096 (8)	0.085 (7)	0.053 (6)	−0.027 (6)	0.002 (5)	0.000 (6)
C16	0.069 (6)	0.073 (6)	0.066 (6)	−0.016 (5)	0.004 (5)	0.018 (5)
C17	0.051 (4)	0.050 (4)	0.045 (4)	−0.008 (4)	0.011 (3)	0.007 (4)
C18	0.051 (5)	0.071 (5)	0.061 (5)	−0.003 (4)	0.016 (4)	0.004 (4)
C19	0.068 (6)	0.102 (7)	0.071 (7)	0.000 (6)	0.032 (5)	0.009 (6)
C20	0.090 (8)	0.101 (8)	0.060 (6)	0.009 (6)	0.028 (5)	−0.011 (6)
C21	0.078 (7)	0.086 (6)	0.062 (6)	−0.012 (5)	0.021 (5)	−0.017 (5)
C22	0.059 (5)	0.075 (5)	0.061 (5)	−0.007 (4)	0.018 (4)	−0.004 (5)
C23	0.049 (5)	0.043 (4)	0.062 (5)	0.003 (3)	0.017 (4)	0.007 (4)
C24	0.064 (6)	0.057 (5)	0.081 (6)	−0.008 (4)	0.015 (5)	0.004 (5)
C25	0.081 (7)	0.050 (5)	0.108 (8)	−0.010 (5)	0.028 (6)	0.004 (5)
C26	0.064 (6)	0.061 (6)	0.115 (9)	−0.013 (5)	0.008 (6)	−0.017 (6)
C27	0.060 (5)	0.063 (5)	0.096 (7)	0.000 (4)	−0.010 (5)	−0.019 (5)
C28	0.058 (5)	0.055 (5)	0.073 (6)	0.000 (4)	0.003 (4)	−0.010 (4)
C29	0.055 (5)	0.048 (4)	0.048 (5)	−0.003 (4)	0.012 (4)	0.012 (4)
C30	0.069 (6)	0.074 (6)	0.064 (6)	0.011 (5)	0.014 (5)	0.010 (5)
C31	0.083 (7)	0.092 (7)	0.081 (7)	0.025 (6)	0.018 (6)	0.012 (6)
C32	0.075 (7)	0.098 (8)	0.101 (9)	0.026 (6)	0.007 (7)	0.022 (7)
C33	0.060 (6)	0.105 (8)	0.074 (7)	0.004 (5)	0.004 (5)	0.029 (6)
C34	0.058 (5)	0.068 (5)	0.059 (6)	0.003 (4)	0.005 (4)	0.023 (4)
C35	0.074 (6)	0.068 (5)	0.053 (6)	−0.024 (5)	−0.009 (5)	0.008 (4)
C36	0.099 (8)	0.090 (7)	0.069 (7)	−0.036 (6)	−0.007 (6)	0.009 (5)
C37	0.145 (12)	0.140 (10)	0.092 (10)	−0.076 (9)	−0.018 (9)	−0.001 (8)
C38	0.170 (14)	0.156 (12)	0.080 (10)	−0.072 (11)	−0.033 (10)	−0.002 (9)
C39	0.161 (12)	0.146 (10)	0.063 (7)	−0.065 (10)	−0.021 (8)	0.015 (7)
C40	0.119 (8)	0.093 (7)	0.054 (6)	−0.043 (6)	−0.007 (6)	0.009 (6)
C41	0.043 (4)	0.050 (4)	0.061 (5)	−0.010 (3)	0.008 (4)	0.009 (4)
C42	0.055 (5)	0.056 (5)	0.072 (6)	0.001 (4)	0.014 (4)	0.008 (4)
C43	0.077 (7)	0.058 (5)	0.077 (6)	−0.006 (5)	0.036 (5)	−0.003 (5)
C44	0.071 (7)	0.059 (5)	0.108 (9)	−0.009 (5)	0.050 (6)	−0.008 (6)
C45	0.047 (6)	0.058 (5)	0.116 (10)	−0.001 (4)	0.018 (6)	0.008 (5)
C46	0.056 (5)	0.054 (4)	0.081 (6)	−0.006 (4)	0.007 (5)	0.010 (4)
C47	0.054 (5)	0.053 (4)	0.041 (4)	−0.012 (4)	0.003 (4)	0.006 (3)
C48	0.067 (6)	0.061 (5)	0.052 (5)	0.002 (4)	0.005 (4)	0.014 (4)
C49	0.064 (6)	0.082 (6)	0.061 (6)	−0.010 (5)	0.020 (4)	0.017 (5)
C50	0.083 (7)	0.075 (6)	0.067 (6)	−0.031 (5)	0.017 (5)	0.013 (5)
C51	0.081 (7)	0.054 (5)	0.077 (6)	−0.018 (5)	0.015 (5)	0.000 (4)
C52	0.060 (5)	0.056 (5)	0.061 (5)	−0.008 (4)	0.013 (4)	0.005 (4)

### Geometric parameters (Å, °)

**Table:**

Cu1—N1	2.095 (7)	C22—H22	0.9300
Cu1—N2	2.178 (6)	C23—C28	1.379 (10)
Cu1—P2	2.2648 (19)	C23—C24	1.381 (10)
Cu1—P1	2.276 (2)	C24—C25	1.412 (12)
Br1—C14	1.904 (10)	C24—H24	0.9300
F1—B1	1.361 (16)	C25—C26	1.347 (13)
F2—B1	1.402 (18)	C25—H25	0.9300
F3—B1	1.334 (16)	C26—C27	1.359 (13)
F4—B1	1.250 (18)	C26—H26	0.9300
N1—C1	1.320 (10)	C27—C28	1.386 (11)
N1—C5	1.335 (11)	C27—H27	0.9300
N2—C6	1.338 (10)	C28—H28	0.9300
N2—C10	1.356 (10)	C29—C34	1.392 (10)
P1—C23	1.816 (7)	C29—C30	1.395 (11)
P1—C17	1.825 (8)	C30—C31	1.348 (12)
P1—C29	1.828 (7)	C30—H30	0.9300
P2—C41	1.810 (8)	C31—C32	1.353 (14)
P2—C47	1.817 (7)	C31—H31	0.9300
P2—C35	1.832 (8)	C32—C33	1.370 (14)
C1—C2	1.407 (13)	C32—H32	0.9300
C1—H1	0.9300	C33—C34	1.404 (12)
C2—C3	1.277 (14)	C33—H33	0.9300
C2—H2	0.9300	C34—H34	0.9300
C3—C4	1.369 (15)	C35—C36	1.370 (12)
C3—H3	0.9300	C35—C40	1.375 (13)
C4—C5	1.396 (13)	C36—C37	1.367 (14)
C4—H4	0.9300	C36—H36	0.9300
C5—C6	1.472 (12)	C37—C38	1.39 (2)
C6—C7	1.391 (12)	C37—H37	0.9300
C7—C8	1.343 (14)	C38—C39	1.350 (18)
C7—H7	0.9300	C38—H38	0.9300
C8—C9	1.360 (14)	C39—C40	1.377 (13)
C8—H8	0.9300	C39—H39	0.9300
C9—C10	1.361 (12)	C40—H40	0.9300
C9—H9	0.9300	C41—C42	1.390 (11)
C10—C11	1.476 (12)	C41—C46	1.415 (10)
C11—C12	1.383 (11)	C42—C43	1.374 (12)
C11—C16	1.408 (12)	C42—H42	0.9300
C12—C13	1.366 (11)	C43—C44	1.348 (12)
C12—H12	0.9300	C43—H43	0.9300
C13—C14	1.388 (12)	C44—C45	1.383 (13)
C13—H13	0.9300	C44—H44	0.9300
C14—C15	1.346 (12)	C45—C46	1.362 (13)
C15—C16	1.357 (13)	C45—H45	0.9300
C15—H15	0.9300	C46—H46	0.9300
C16—H16	0.9300	C47—C48	1.391 (11)
C17—C18	1.379 (11)	C47—C52	1.393 (11)
C17—C22	1.384 (10)	C48—C49	1.375 (11)
C18—C19	1.390 (12)	C48—H48	0.9300
C18—H18	0.9300	C49—C50	1.372 (12)
C19—C20	1.390 (13)	C49—H49	0.9300
C19—H19	0.9300	C50—C51	1.341 (13)
C20—C21	1.337 (13)	C50—H50	0.9300
C20—H20	0.9300	C51—C52	1.386 (11)
C21—C22	1.370 (12)	C51—H51	0.9300
C21—H21	0.9300	C52—H52	0.9300
			
N1—Cu1—N2	78.7 (3)	C21—C22—C17	123.0 (8)
N1—Cu1—P2	105.7 (2)	C21—C22—H22	118.5
N2—Cu1—P2	126.24 (16)	C17—C22—H22	118.5
N1—Cu1—P1	107.35 (18)	C28—C23—C24	118.7 (7)
N2—Cu1—P1	98.60 (16)	C28—C23—P1	118.6 (6)
P2—Cu1—P1	128.29 (9)	C24—C23—P1	122.8 (6)
C1—N1—C5	119.2 (8)	C23—C24—C25	119.2 (8)
C1—N1—Cu1	127.1 (7)	C23—C24—H24	120.4
C5—N1—Cu1	113.7 (6)	C25—C24—H24	120.4
C6—N2—C10	117.3 (7)	C26—C25—C24	120.2 (9)
C6—N2—Cu1	110.4 (5)	C26—C25—H25	119.9
C10—N2—Cu1	128.5 (6)	C24—C25—H25	119.9
C23—P1—C17	103.0 (4)	C25—C26—C27	121.4 (9)
C23—P1—C29	101.9 (3)	C25—C26—H26	119.3
C17—P1—C29	109.0 (3)	C27—C26—H26	119.3
C23—P1—Cu1	111.5 (3)	C26—C27—C28	118.8 (9)
C17—P1—Cu1	119.0 (2)	C26—C27—H27	120.6
C29—P1—Cu1	110.8 (2)	C28—C27—H27	120.6
C41—P2—C47	104.7 (4)	C23—C28—C27	121.6 (8)
C41—P2—C35	104.0 (4)	C23—C28—H28	119.2
C47—P2—C35	103.5 (4)	C27—C28—H28	119.2
C41—P2—Cu1	119.2 (3)	C34—C29—C30	118.7 (7)
C47—P2—Cu1	112.2 (3)	C34—C29—P1	123.4 (6)
C35—P2—Cu1	111.7 (3)	C30—C29—P1	117.7 (6)
F4—B1—F3	116.7 (15)	C31—C30—C29	120.0 (9)
F4—B1—F1	113.7 (14)	C31—C30—H30	120.0
F3—B1—F1	110.0 (14)	C29—C30—H30	120.0
F4—B1—F2	107.0 (15)	C30—C31—C32	123.2 (10)
F3—B1—F2	102.5 (13)	C30—C31—H31	118.4
F1—B1—F2	105.7 (12)	C32—C31—H31	118.4
N1—C1—C2	122.3 (10)	C31—C32—C33	118.0 (9)
N1—C1—H1	118.8	C31—C32—H32	121.0
C2—C1—H1	118.8	C33—C32—H32	121.0
C3—C2—C1	117.8 (11)	C32—C33—C34	121.5 (9)
C3—C2—H2	121.1	C32—C33—H33	119.2
C1—C2—H2	121.1	C34—C33—H33	119.2
C2—C3—C4	122.6 (11)	C29—C34—C33	118.5 (9)
C2—C3—H3	118.7	C29—C34—H34	120.7
C4—C3—H3	118.7	C33—C34—H34	120.7
C3—C4—C5	118.3 (11)	C36—C35—C40	118.4 (8)
C3—C4—H4	120.8	C36—C35—P2	119.7 (8)
C5—C4—H4	120.8	C40—C35—P2	121.1 (7)
N1—C5—C4	119.7 (9)	C37—C36—C35	123.0 (12)
N1—C5—C6	117.7 (8)	C37—C36—H36	118.5
C4—C5—C6	122.5 (10)	C35—C36—H36	118.5
N2—C6—C7	122.4 (9)	C36—C37—C38	116.2 (12)
N2—C6—C5	116.7 (8)	C36—C37—H37	121.9
C7—C6—C5	120.7 (9)	C38—C37—H37	121.9
C8—C7—C6	118.9 (10)	C39—C38—C37	121.9 (11)
C8—C7—H7	120.6	C39—C38—H38	119.0
C6—C7—H7	120.6	C37—C38—H38	119.0
C7—C8—C9	119.5 (10)	C38—C39—C40	120.1 (13)
C7—C8—H8	120.2	C38—C39—H39	120.0
C9—C8—H8	120.2	C40—C39—H39	120.0
C8—C9—C10	120.1 (10)	C35—C40—C39	119.7 (10)
C8—C9—H9	119.9	C35—C40—H40	120.1
C10—C9—H9	119.9	C39—C40—H40	120.1
N2—C10—C9	121.8 (9)	C42—C41—C46	116.7 (8)
N2—C10—C11	117.9 (7)	C42—C41—P2	119.0 (6)
C9—C10—C11	120.3 (9)	C46—C41—P2	124.3 (7)
C12—C11—C16	116.4 (8)	C43—C42—C41	120.8 (8)
C12—C11—C10	123.4 (8)	C43—C42—H42	119.6
C16—C11—C10	120.0 (8)	C41—C42—H42	119.6
C13—C12—C11	122.4 (8)	C44—C43—C42	122.5 (10)
C13—C12—H12	118.8	C44—C43—H43	118.7
C11—C12—H12	118.8	C42—C43—H43	118.7
C12—C13—C14	118.7 (8)	C43—C44—C45	117.6 (9)
C12—C13—H13	120.6	C43—C44—H44	121.2
C14—C13—H13	120.6	C45—C44—H44	121.2
C15—C14—C13	120.5 (9)	C46—C45—C44	122.0 (8)
C15—C14—Br1	121.3 (8)	C46—C45—H45	119.0
C13—C14—Br1	118.2 (8)	C44—C45—H45	119.0
C14—C15—C16	120.7 (9)	C45—C46—C41	120.4 (9)
C14—C15—H15	119.6	C45—C46—H46	119.8
C16—C15—H15	119.6	C41—C46—H46	119.8
C15—C16—C11	121.3 (9)	C48—C47—C52	117.9 (7)
C15—C16—H16	119.4	C48—C47—P2	119.7 (6)
C11—C16—H16	119.4	C52—C47—P2	122.3 (6)
C18—C17—C22	116.9 (8)	C49—C48—C47	122.1 (8)
C18—C17—P1	123.1 (6)	C49—C48—H48	119.0
C22—C17—P1	119.8 (6)	C47—C48—H48	119.0
C17—C18—C19	120.9 (8)	C50—C49—C48	117.9 (8)
C17—C18—H18	119.6	C50—C49—H49	121.0
C19—C18—H18	119.6	C48—C49—H49	121.0
C18—C19—C20	119.1 (9)	C51—C50—C49	122.0 (8)
C18—C19—H19	120.5	C51—C50—H50	119.0
C20—C19—H19	120.5	C49—C50—H50	119.0
C21—C20—C19	121.0 (10)	C50—C51—C52	120.6 (9)
C21—C20—H20	119.5	C50—C51—H51	119.7
C19—C20—H20	119.5	C52—C51—H51	119.7
C20—C21—C22	119.0 (9)	C51—C52—C47	119.6 (9)
C20—C21—H21	120.5	C51—C52—H52	120.2
C22—C21—H21	120.5	C47—C52—H52	120.2

### Hydrogen-bond geometry (Å, °)

**Table:**

*D*—H···*A*	*D*—H	H···*A*	*D*···*A*	*D*—H···*A*
C26—H26···F2	0.93	2.53	3.185 (12)	127.
C27—H27···F3	0.93	2.48	3.356 (11)	158.
